# DNA isolation and genome sequence of the 134‐year‐old holotype specimen of *Boletus subvelutipes* Peck

**DOI:** 10.1002/ece3.10389

**Published:** 2023-08-10

**Authors:** Maria Shumskaya, Kirill S. Mironov, Jesús A. Ballesteros, Igor Safonov, Roy E. Halling

**Affiliations:** ^1^ Department of Biology, CSMT Kean University Union New Jersey USA; ^2^ Department of Molecular Biosystems K.A. Timiryazev Institute of Plant Physiology Moscow Russian Federation; ^3^ The New Jersey Mycological Association Maple Shade New Jersey USA; ^4^ The New York Botanical Garden Institute of Systematic Botany Bronx New York USA

**Keywords:** Boletaceae, fungi, herbarium specimens, historic DNA, holotype genome, museomics

## Abstract

Molecular characterization of type specimens is a powerful tool used in clarifying species identity/circumscription, as well as establishing the taxonomic and phylogenetic status of organisms in question. However, DNA sequencing of aged herbarium collections can be a challenge due to the quantity and quality of DNA still present in the specimens. Herein, we report a custom DNA isolation protocol suitable for processing minute quantities of old specimen tissue and its utilization via high‐throughput sequencing technologies to obtain, for the first time, the genome assembly of the 134‐year‐old holotype of *Boletus subvelutipes* Peck, a North American fleshy pored mushroom of taxonomic and historical significance. A side‐by‐side evaluation of our DNA isolation method with that of a commercial “kit” by Qiagen is also presented. By relying on the type material, we have established the genetic identity of *B. subvelutipes*, as well as providing preliminary phylogenetic evidence for its generic affinities in *Neoboletus* within Boletaceae. The reference genome of the *B. subvelutipes* holotype provides a resource for future comparative genomic studies, taxonomic revisions in Boletaceae, and other evolutionary studies of fungi.

## 1 INTRODUCTION

Conventional classification of fungi used to rely exclusively on gross morphology, with microscopic examination of the holotype material often required to clarify taxonomic boundaries. Rapid developments in DNA analysis and ancillary technologies have resulted in the revision of fungal phylogeny (Hibbett et al., 2007). Such studies employ DNA barcoding to classify fungal taxa (Eberhardt, 2012; Horn et al., 2020; Kress & Erickson, 2012; Schoch et al., 2012; Xu, 2016), with the barcodes being genomic regions of ITS (Internal Transcribed Spacer), TEF1 (translation elongation factor 1), LSU and SSU (the large and small ribosome subunits, respectively), and others (Bridge et al., 2005; Zhang et al., 2017). However, DNA sequencing of historical/older herbarium exsiccatae can be challenging. Because of the intrinsic value of holotype specimens as references, access to and destructive sampling of museum material are often restricted; furthermore, contaminated and highly fragmented DNA of aged and manipulated collections can be difficult to amplify. For example, DNA barcoding of the ITS region of fungi requires 550 bp on average (Nilsson et al., 2015), which is hard to obtain intact from aged samples.

**FIGURE 1:**
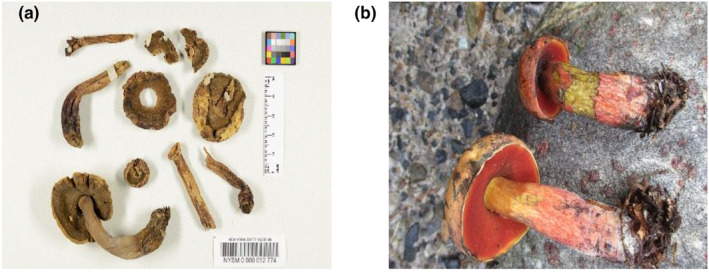
(a) *Boletus subvelutipes* voucher NYSM:NYSf3093, New York State Museum Herbarium (photo by Dr. Lorinda Leonardi). (b) A modern collection of *B. subvelutipes* voucher MO285181 from type locality near Caroga Lake, NY (photo by David H. Wasilewski).

Here, we present a method of DNA isolation culminating in a successful genome sequencing of the 134‐year‐old holotype specimen of *Boletus subvelutipes* Peck (Figure 1a)—one of the first described North American boletes featuring a red pore surface and oxidative bluing (Figure 1b; Peck, 1889). Collected near Caroga Lake, Fulton Co., New York, in July 1888, the holotype specimen is currently stored at the New York State Museum (NYSM) herbarium, where Peck himself originally deposited it (voucher NYSM:NYSf3093, Figure 1a).

Charles Peck contributed monumentally to the mycological legacy in North America from 1867 to 1915, during which time he described 2734 species (Gilbertson, 1962; Haines, 1986). The clarification of Peck's taxonomic concepts for North American fungi using DNA sequencing technology has had significant global impact on many groups of macrofungi (Delgat et al., 2019; Kuo et al., 2012).

In 1909, Murrill transferred Peck's bolete to *Suillellus*, a new genus, with *Boletus luridus* as the generic type species (Murrill, 1909). Later, *Boletus subvelutipes* was placed in *Boletus* subsect. *Luridi* Fr., based on gross morphology (Smith & Thiers, 1971). This species may have undergone a complex taxonomic history in the presence of morphologically similar taxa subsequently published by other mycologists (Both, 1993).

However, *Boletus* sensu *lato* is not monophyletic (Nuhn et al., 2013). Once a very large genus (Singer, 1986; Smith & Thiers, 1971; Snell & Dick, 1970), *Boletus* of today is narrowly circumscribed by porcini mushrooms, that is, *Boletus edulis* and allies (Dentinger et al., 2010), hence *B. subvelutipes* placement in *Boletus* is unlikely.

The problem of identification of taxa remaining in *Boletus* sensu *lato* continues to be fueled by taxonomic uncertainties (Smith & Thiers, 1971; Treu, 1994) due to the lack of published DNA data from type collections. Recent molecular studies place the vast majority of red‐pored *Boleti* in *Neoboletus*, *Suillellus*, or *Rubroboletus* (Vizzini et al., 2014). Morphologically, *B. subvelutipes* does not appear to fit the generic concept of *Rubroboletus* (Zhao et al., 2014) because many representatives of this genus have prominently reticulated stipes, for example, *R. rhodosanguineus* from eastern North America; thus its placement into *Neoboletus* or *Suillellus* is more likely.

The reference genome of the *B. subvelutipes* holotype presented in this paper provides a resource for future comparative genomic studies, taxonomic revisions in Boletaceae, and other evolutionary studies of fungi.

## 2 MATERIALS AND METHODS

### 2.1 DNA isolation

Approximately 5 mm^3^ of dry tissue of *B. subvelutipes* holotype, collection number NYSf3093, was received from the New York State Museum herbarium, Albany, NY, USA. The tissue was split into two parts, each part transferred to a 2 mL 1.5 mm triple‐pure zirconium beads tube (Benchmark Scientific); half of the standard bead load was used. 800 μL of water was added to each tube and then the tubes were homogenized by BeadBug (Benchmark Scientific), 180 s, maximum speed. The tubes were centrifuged for 1 min at 14,000 *g*, and the supernatants were transferred to fresh 2 mL Eppendorf tubes. The solutions in each tube were then subjected to either “manual” or “kit” purification protocol to purify DNA.

1. Protocol “manual”: the supernatant was mixed with 800 μL of 20% Chelex‐100 beads in water (BioRad) and 100 μL of proteinase K (Promega). The sample was incubated overnight at 55°C with occasional shaking. After that, the sample was incubated for 10 min at 95°C to inactivate proteinase K, and centrifuged for 5 min at 14,000 *g* to precipitate Chelex beads. The resultant supernatant was transferred to Amicon Ultra‐0.5 30K Centrifugal Filters (Millipore Sigma), centrifuged 10 min 14,000 *g*, and then washed with 500 μL of Tris–HCl pH 8.0 and centrifuged again using the same settings, with the flow‐through discarded. The resultant concentrated 25 μL of DNA was mixed with 1.8 volume (45 μL) of AMPure XP magnetic beads (Beckman Coulter), and processed according to the manufacturer's instructions. The DNA was eluted from the beads using 25 μL of Tris–HCl pH 8.0 and stored at −80°C.
2. Protocol “kit”: the supernatant was subjected to DNA extraction using QIAamp DNA Micro kit (Qiagen), with “Isolation of Genomic DNA from Tissues” protocol supplied by the manufacturer. In short, the supernatant was treated with a provided proteinase K solution, then together with the carrier RNA loaded to QIAamp MiniElute column, washed with provided washing buffers, and eluted from the column with 50 μL of Tris–HCl pH 8.0, then stored at −80°C.

The concentration of purified dsDNA was measured by Qubit 2.0 Fluorometer (Life Technologies) using dsDNA HS kit and by NanoDrop 2000 (Thermo Scientific).

### 2.2 DNA sequencing and analysis

The DNA amplicon library preparation and sequencing reactions were conducted at Genewiz/Azenta (South Plainfield) as follows:

NEBNext® Ultra™ DNA Library Prep Kit for Illumina (New England Biolabs), clustering, and sequencing reagents were used following the manufacturer's recommendations. Briefly, fragmented DNA was end‐repaired and 3′‐adenylated. After adenylation, the adapters were ligated and subjected to enrichment by limited cycle PCR. The DNA library was validated using D1000 ScreenTape on the Agilent 4200 TapeStation (Agilent Technologies), and quantified using Qubit. The DNA library was quantified by real‐time PCR (Applied Biosystems), clustered in a flow cell, and loaded on Illumina MiSeq instrument according to manufacturer's instructions. The sample was sequenced using a 2 × 150 paired‐end (PE) configuration. Image analysis and base calling were performed by MiSeq Control Software (MCS). Raw sequencing data (.bcl files) was converted into fastq files and de‐multiplexed using Illumina's bcl2fastq v.2.20 software. One mismatch was allowed for index sequence identification.

The Illumina adaptor sequences were removed prior to the genome assembly step. Specifically, the adaptor 3′‐sequences were deleted using Trim Galore v.0.6.5 https://github.com/FelixKrueger/TrimGalore in the paired‐end read mode with default parameters. Kraken 2 (v. 2.1.2) was used to search for contaminating sequences in the read files (Lu et al., 2022; Wood et al., 2019). PlusPFP‐index with construction date 3/14/2023 https://benlangmead.github.io/aws‐indexes/k2 was used as the database for read classification.

The genome size was estimated with k‐mer analysis performed using kmercountexact.sh from BBMap—Bushnell B.—sourceforge.net/projects/bbmap/. Then, contiguous genomic regions were assembled from the DNA reads. The contigs were assembled de novo using the SPAdes v.3.15.3 (Prjibelski et al., 2020) genomic assembler with the flag “‐‐isolate” and k‐mer length values of 21, 25, 33, 55, 77, 99, 111, and 127. *N*_50_ and other metrics were built with QUAST v.5.0.2 (Gurevich et al., 2013). Following this, the contigs were analyzed for the presence of contaminants from nonfungal origin, such as human, plant, and bacterial DNA. BWA v.0.7.17 (Li & Durbin, 2009, 2010) and SAMtools v.1.10 (Li et al., 2009) were used to align‐back reads to NYSf3093 and MG31 assemblies for coverage analysis, and to align to hg38 DNA. Removal of human DNA contamination was performed by aligning contigs to the hg38 sequence using the BWA algorithm, after which unaligned sequences were filtered out using SAMtools before submission to NCBI. Additional filtering was performed by NCBI algorithms and the NCBI‐cleaned assembly was used for the subsequent analysis. Blobtools v.1.1.1 was also used for the analysis of possible genome contamination according to the developers recommendations https://blobtools.readme.io/docs (Laetsch & Blaxer, 2017).

The contigs were also analyzed with BUSCO v.5.4.4 (Manni et al., 2021) in the genome mode using boletales_odb10 as database.

For the phylogenetic analysis, specific sequences used in fungal phylogeny were extracted from the genome and saved separately. NCBI‐BLAST v.2.12.0 blastn (Altschul et al., 1990) was used to search the genome for fragments of mitochondrial genome, rRNA‐encoding regions, TEF1, RPB2, and atp6 genes; some of these loci were then used to construct the phylogeny.

### 2.3 Genome annotation and analysis

In order to assign genes with possible functions, reference odb10_fungi protein sequences were downloaded from https://v100.orthodb.org/download/odb10_fungi_fasta.tar.gz and prepared according to https://github.com/gatech‐genemark/ProtHint#protein‐database‐preparation. The script braker.pl (Brůna et al., 2020, 2021; Gotoh, 2008; Hoff et al., 2016, 2019; Iwata & Gotoh, 2012; Lomsadze et al., 2005; Stanke et al., 2006, 2008; Ter‐Hovhannisyan et al., 2008) was run in “BRAKER with proteins of any evolutionary distance” mode with a flag “—fungus.” BUSCO v.5.2.2 (Manni et al., 2021) was run in a protein mode using boletales_odb10 as the database. dbCAN2 meta‐server (https://bcb.unl.edu/dbCAN2; Zhang et al., 2018) was used to search and analyze enzymes from the CAZymes group, a family of proteins characteristic of fungi. SignalP v.6.0 was used for secretory proteins search in the CAZymes supergroup (Teufel et al., 2022).

### 2.4 Phylogenetic analysis

Phylogenetic analysis was performed to infer the placement *B. subvelutipes* NYSf3093 within Boletaceae. Multilocus phylogeny taxa and locus selection followed the species published in Chai et al. (2019). GenBank accession numbers for the backbone phylogeny are presented in Data S1. Homologous regions of ITS, 28S, and TEF1 were identified and extracted from *B. subvelutipes* MG31 (ASM331603, GCA_003316035.1) and NYSf3093 (JAMFLD000000000) by BLASTn search of ITS, 28S, and TEF1 from *B. edulis* (Boled5) against ASM331603‐ and JAMFLD000000000‐databases constructed with makeblastdb v. 2.13.0+. For RPB2, the *B. edulis* sequence (ADK11879) was downloaded from NCBI and used as a query sequence for DIAMOND‐alignment (Buchfink et al., 2015) against the amino acid sequences MG31‐braker and NYSf3093‐braker generated by BRAKER (Hoff et al., 2019). The found sequences with *e*‐value = 0 and identity close to 100% were considered to be target genes. Only taxa with at least 3 of the 4 available loci were carried out for downstream phylogenetic analyses, resulting in a final matrix with 96 taxa. The ITS and 28S loci were aligned with MAFFT (v7.490, mafft ‐‐auto) (Katoh & Standley, 2013), and gap rich sites were removed from the alignment using TrimAl v1.2rev59 (‐gt 0.75) (Capella‐Gutierrez et al., 2009). Protein coding loci were aligned with MACSE v2.06 (Ranwez et al., 2018) preserving the open reading frame. Loci were concatenated with geneStitcher.py (Ballesteros & Hormiga, 2016) and analyzed with IQ‐TREE (v1.6.12, ‐‐m MFP + Merge ‐bb 1000 ‐bnni) for finding best substitution and partition merging strategy, followed by maximum likelihood analyses with 1000 ultrafast bootstraps for estimate branch support (Chernomor et al., 2016; Hoang et al., 2018; Kalyaanamoorthy et al., 2017; Nguyen et al., 2015). The model finder found the best partition and substitution model with five different partitions: 28S (TNe + R3), ITS (K2P + I + G4), 1st codon TEF1 + 1st and 2nd codon rpb2 (K2P + I + G4), tef1 2nd codon (TIMe + G4) and TEF1 3rd codon + rpb2 3rd codon (HKY + F + I + G4).

## 3 RESULTS AND DISCUSSION

### 3.1 DNA isolation and sequencing

DNA isolation from historic samples is always a challenge (Burrell et al., 2015). Common genomic DNA isolation protocols include DNA purification steps, such as purification of DNA via phenol:chlorophorm:isoamyl alcohol mix in “in‐house” protocols, or filtration of DNA through a mini‐column in commercial kits, which contains a filter that specifically binds to nucleic acids and allows for washing with buffers to remove proteins and other impurities; both result in a significant loss of DNA. Small DNA fragments are not retained by the filter; also, micro amounts of DNA in commercial protocols often require carrier RNA to be able to bind to the filter. With museum samples, even minimal DNA loss may be critical, resulting in genetic material that is impossible to work with using standard DNA‐barcoding methods. This is related to the fact that storage history of old herbarium specimens is often unclear, and poor storage conditions and contamination by environmental and human DNA reduce the quality of isolatable genetic material from dated fungal samples. Overdrying, aging, and prolonged exposure to harsh pest control agents, such as arsenic and naphthalene used in the late 1800s, can result in degraded genomic DNA, or its very small amount that the researcher cannot afford to lose.

**FIGURE 2:**
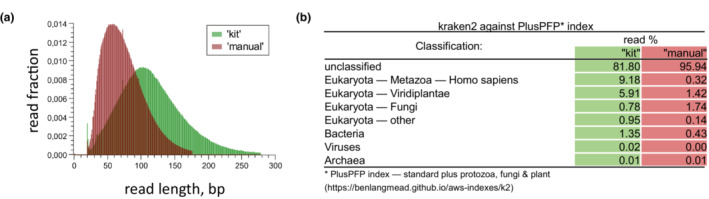
Comparison of two methods of genomic DNA isolation. “kit”—DNA isolated using the “kit” protocol; “manual”—DNA isolated using the “manual” protocol. (a) Average size of DNA inserts (bp) between Illumina adapters in samples of genomic DNA isolated using “kit” vs. “manual” methods. (b) Kraken2 classification of the “kit” and “manual” reads using PlusPFP‐index that contained standard plus protozoa, fungi & plant (date 3/14/2023), https://benlangmead.github.io/aws‐indexes/k2.

In our study, we were given only a 5 mm^3^ piece of herbarium tissue of *B. subvelutipes*, which we used completely, as the herbarium could not provide a larger sample for destruction due to the uniqueness of the specimen. We have split this sample in half to explore two different DNA isolation methods. Our in‐house “manual” method was designed to preserve as much as possible of the native genomic DNA and included tissue homogenization, protein removal with proteinase K in the presence of Chelex‐100 to remove Mg^2+^ (an essential cofactor for DNAses), followed by concentration of the genomic DNA X20 using Amicon Ultra‐0.5 30‐K centrifugal concentrators. These concentrators remove from the solution everything of <30 kDa. The concentrated DNA solution of ca 25 μL was then purified using magnetic beads AMPure XP. We have also used a commercial “kit” from Qiagen, specifically designed to isolate micro amounts of DNA. The “kit” included a DNA purification step via a mini‐column and resulted in the isolation of much smaller amounts of DNA, loss of shorter fragments, and a more contaminated DNA as we later confirmed by high‐throughput sequencing (Figure 2, analysis by Kraken 2; Wood et al., 2019). The 260/280‐nm ratio for the DNA isolated using the “manual” method was 1.88, A260/230 = 1.34; for the “kit” method, it was 2.2, A260/230 = 1.21. The high A260/280 ratio for the “kit” method is a result of the presence of carrier RNA in the purification protocol, a necessary component of the kit.

Our initial attempts to perform DNA barcoding using standard procedures described earlier (Eberhardt, 2012; Schoch et al., 2012) with our extracted DNA were unsuccessful. We were not able to amplify any parts of ITS with various combinations of ITS‐specific primers, although PCR conditions were modified according to studies of old herbarium samples (Larsson & Jacobsson, 2004). At this point, the classic DNA‐barcoding technique would be deemed unsuitable for this specimen and the molecular work would cease. Following this failure, we proceeded to sequence the DNA isolated by both methods using the short read whole genome sequencing (WGS) with Illumina 2 × 150 bp protocol, and it turned out to be a success. Once the genome was assembled, the sizes of inserts were compared and fragments from the “kit” isolation were found to be mostly of 100 bp, while the “manual” method allowed recovery of 50–70 bp inserts (Figure 2a); this implies that the standard PCR techniques failed due to severe DNA fragmentation. Kraken 2 analysis results (Figure 2b) demonstrates that both DNA samples were contaminated with predominantly human DNA, and the “manual” extraction resulted in less contamination.

### 3.2 Genome assembly and analysis

Sequencing yielded a total of 3.9 × 10^7^ read pairs: 1.9 × 10^7^ for the “kit” and 2.0 × 10^7^ for the “manual” libraries, equivalent to 11.7 Gb in total. 100% of the reads survived the adapter removal procedure. Approximately 30% of these contained deleted adapter sequences, and 1%–3% of the bases were quality‐trimmed. Combining both, the “kit” and “manual” data, we assembled the genome. Then, we aligned the contigs against human hg38‐genome and removed the contigs that were aligned, considering it contamination with human DNA. The remaining (unaligned with hg38) contig set was uploaded to NCBI database (https://www.ncbi.nlm.nih.gov/) as NYSM:NYSf3093 genome, accession number JAMFLD000000000 (hereafter NYSf3093, or holotype assembly). Currently, NCBI contains information about genome ASM331603 (Li et al., 2018), recorded as “*Boletus subvelutipes* strain MG31” (hereafter MG31), and the NCBI server suggests a similarity between our submission and this assembly. The genome size of MG31 is 51.7 Mbp versus 55.6 Mbp for our assembly (Table 1). Our assembly size is acceptable based on the K‐mer analysis, which provides an estimated genome size of 59.4 Mbp for *k* = 63. It is also comparable in size to the genome of another species in Boletaceae, *B. edulis* BED1, whose Boled5 assembly size is 66.5 Mbp (Miyauchi et al., 2020).

**TABLE 1:** Genomic features of the two *Boletus subvelutipes* genome assemblies measured by QUAST.

Metrics	NYSf3093 assembly JAMFLD000000000	MG31 assembly ASM331603
*N* (all contigs)	71,413	15,805
*N* (contigs ≥1000)	12,178	9036
Largest contig	35,544	125,307
*N*_50_	1216	7897
GC%	46.7	48.6
Mbp	55.6	51.7

In order to assess the suitability of MG31 as a reference genome for NYSf3093, and to investigate which DNA isolation method, “kit” or “manual”, produced more suitable for genome assembly reads, we mapped reads obtained from DNA isolated by one or another method onto:

1. NYSf3093 genome to assess which reads were mostly used in the assembly,
2. hg38 to assess with method had more contaminants from human DNA,
3. MG31 genome to assess the similarity of our holotype genome to this species.

Mapping statistics for this analysis is summarized in Table 2.

**TABLE 2:** Mapping statistics of the alignment of DNA reads produced from the two isolation protocols, “kit” and “manual”, onto NYSf3093, hg38, and MG31 genomes.

Genome	DNA reads from the isolation protocol	Mapped %	Mean coverage	Mean quality
NYSf3093	“kit”	87.4	60.6	52.5
	“manual”	94.6	46.7	30.1
hg38	“kit”	78.9	0.3	15.1
	“manual”	57.2	0.2	12.0
MG31	“kit”	50.1	31.4	31.4
	“manual”	41.2	18.7	30.7

We observed that the reads from the “kit”‐isolated DNA had a lower alignment to our holotype NYSf3093 assembly, and a higher proportion of mapped reads onto hg38 (Table 2). Also, we found that the number of alignments to the MG31‐assembly was only 50.1% for the “kit” protocol‐produced reads and 41.2% for the “manual” protocol‐produced reads. Overall, the reads obtained using the “manual” method were characterized by less contamination from hg38 genome and higher percentage of alignments onto the NYSf3093 genome. However, the alignments were of a lower average quality, which can be explained by smaller size of the fragment lengths of the sequencing library. We also concluded that MG31 could not be used as a reference genome for NYSf3093 due to low percent of alignment to MG31 reads.

**FIGURE 3:**
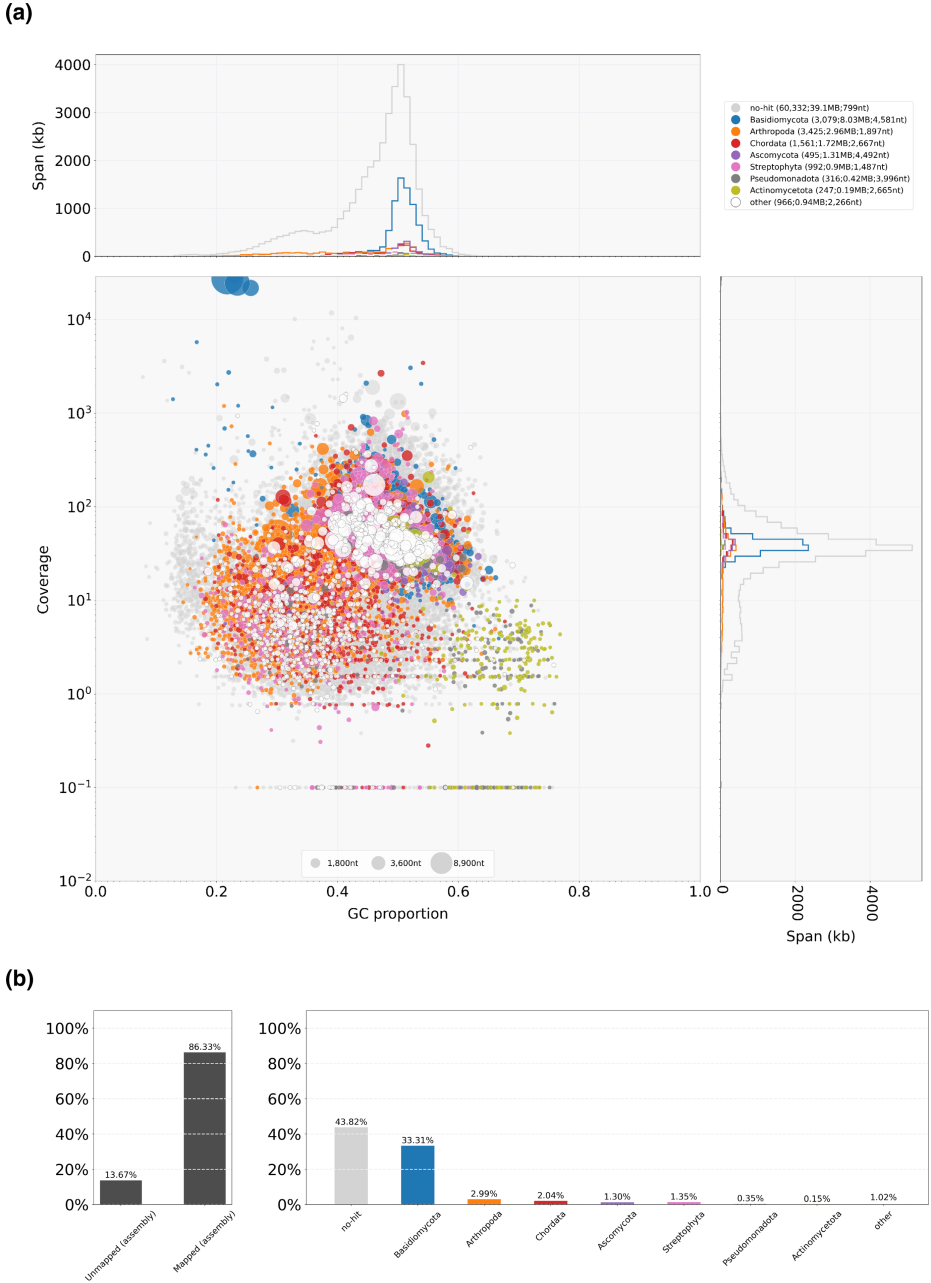
Blobtools analysis of the “kit” reads mapped to the holotype NYSf3093 genome (JAMFLD000000000). NYSf3093 contigs were blast‐searched against nt‐database (06/2023). (a) Taxon‐annotated GC‐coverage plot (blobplot) of “kit” blobDB.json. Each contig is represented by a circle, color corresponding to the taxa in the top right corner. Circles are plotted based on GC content (*x*‐axis) and coverage (*y*‐axis), with a diameter proportional to the length. The top and right histograms summarize contig spans for GC proportion bins and coverage bins, respectively. Top right corner in brackets: number of contigs assigned, total span, *N*_50_ length. (b) “kit” reads coverage of taxonomically classified contigs.

**FIGURE 4:**
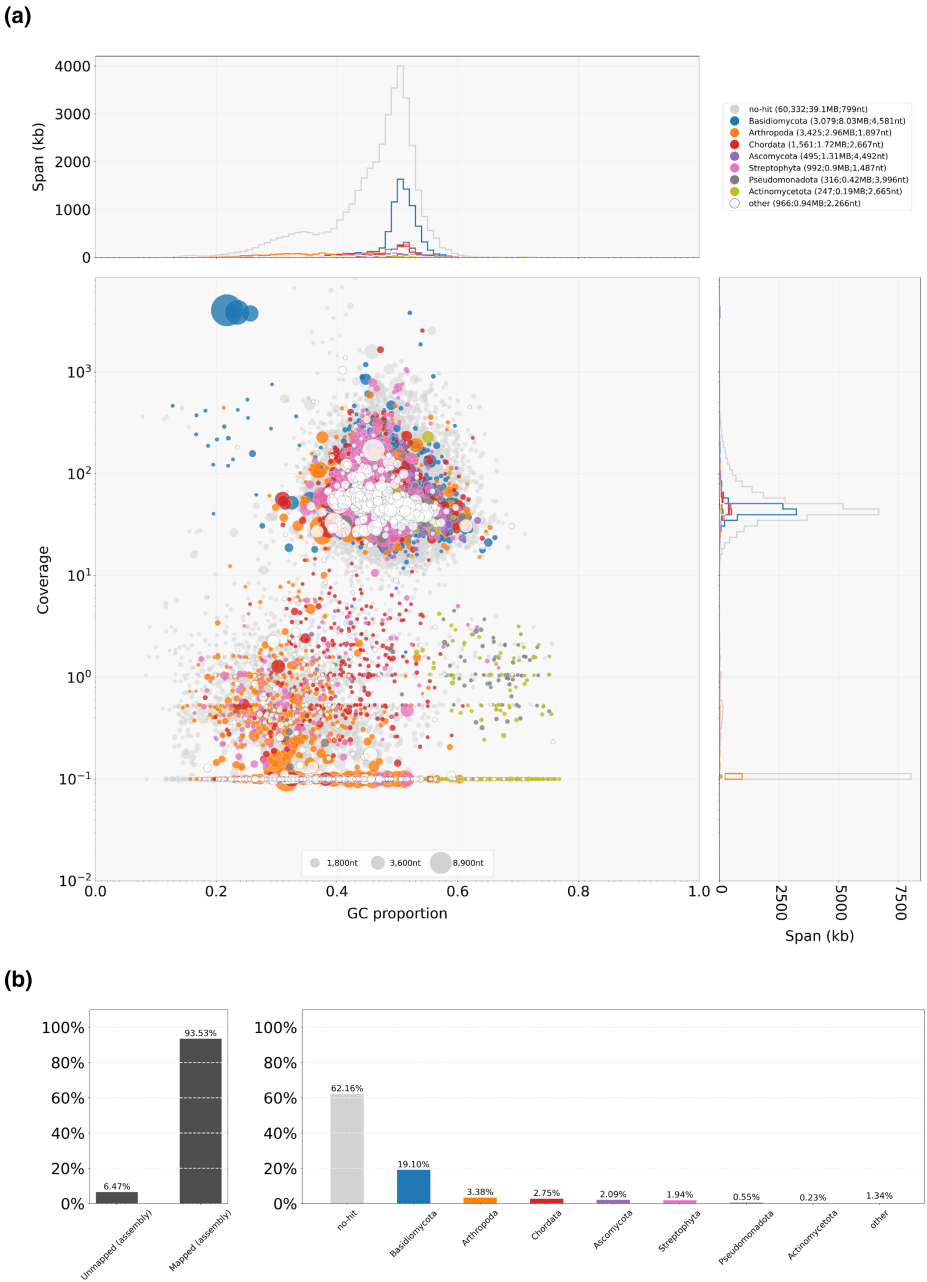
Blobtools analysis results of the “manual” reads mapped to the holotype NYSf3093 genome (JAMFLD000000000). NYSf3093 contigs were blast‐searched against nt‐database (06/2023). (a) Taxon‐annotated GC‐coverage plot (blobplot) of “manual” blobDB.json. Each contig is represented by a circle, color corresponding to the taxa in the top right corner. Circles are plotted based on GC content (*x*‐axis) and coverage (*y*‐axis), with a diameter proportional to the length. The top and right histograms summarize contig spans for GC proportion bins and coverage bins, respectively. Top right corner in brackets: number of contigs assigned, total span, *N*_50_ length. (b) “manual” reads coverage of taxonomically classified contigs.

To assess the quality of the holotype genome in more details, the NYSf3093 assembly was analyzed using a taxon‐annotated GC‐coverage plot in Blobtools pipeline (Laetsch & Blaxer, 2017) (Figures 3 and 4). This analysis confirmed that the genomic DNA isolated by the “manual” protocol was more suitable for the genome assembly, which is shown by the isolated set of NYSf3093 contigs represented by single‐filled circles on the blobplot for the “manual” method (Figure 4a) versus “kit” (Figure 3a).

**FIGURE 5:**
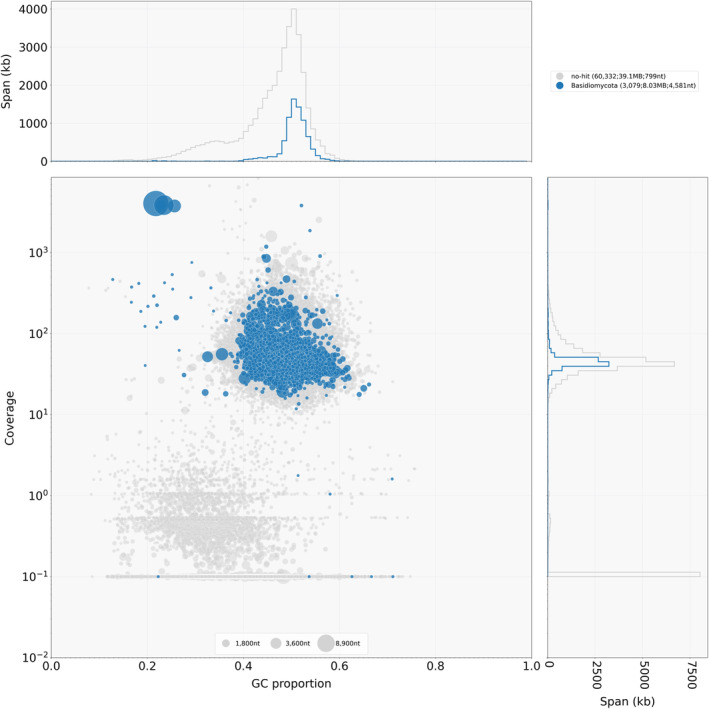
GC‐coverage plot (blobplot) of “manual” blobDB.json plotted for “no‐hit” and “Basidiomycota” contig sets.

Our target genome is likely to be represented by the sets of contigs belonging to the “no‐hit” and “Basidiomycota” groups with “manual” read coverage ≥10^1^. Their corresponding blobplot is given in Figure 5. We also want to emphasize that all the genes used later to construct the phylogeny came from the Basidiomycota contigs set. This set of contigs is characterized by the following quantitative metrics: contigs number is 3079; *N*_50_ = 4581; GC% = 0.5 ± 0.05; total length is 8.0 Mbp.

**FIGURE 6:**
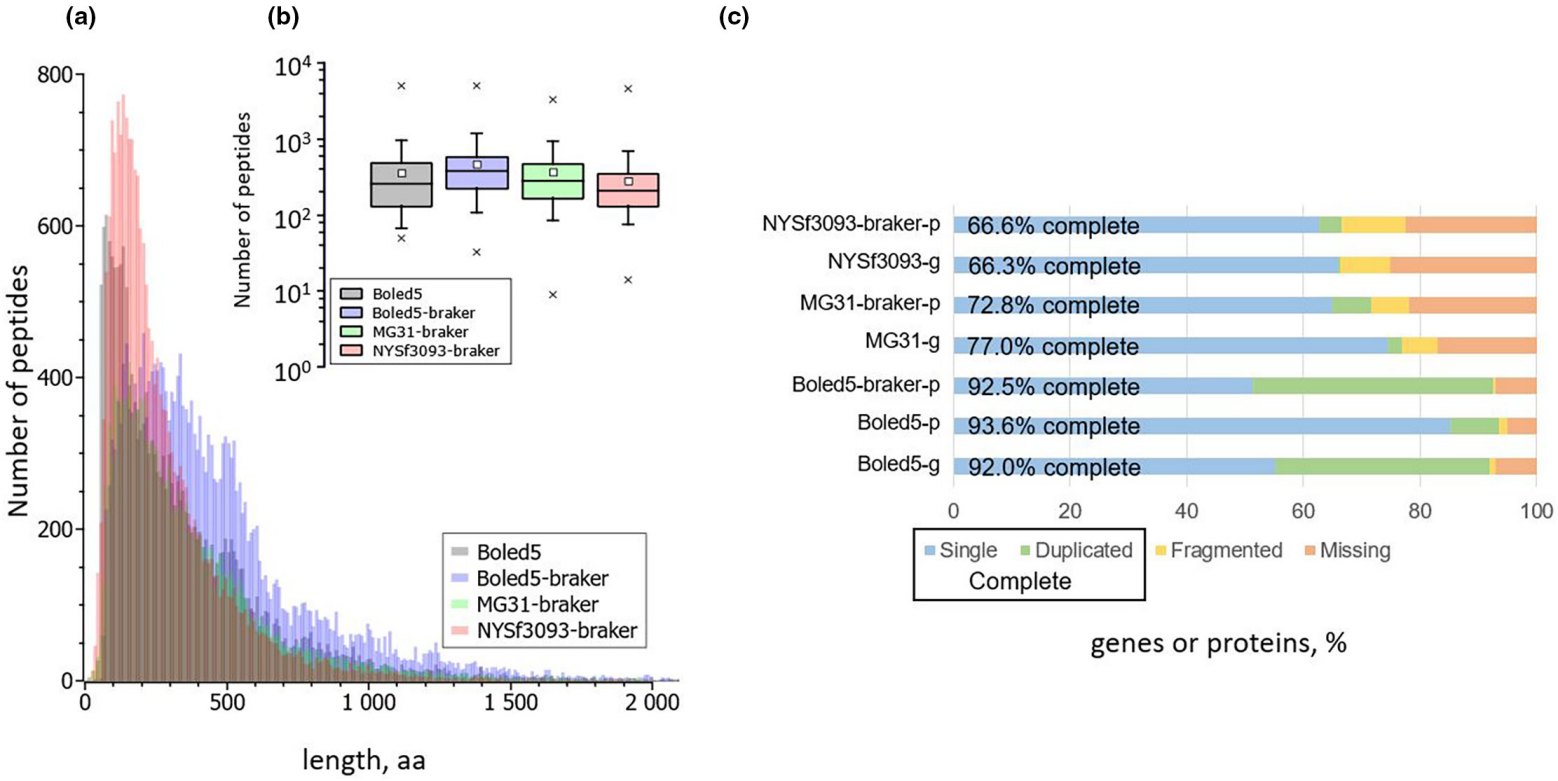
Annotation of NYSf3093 BRAKER. (a) Protein length distribution histogram. (b) Box plot with min–max ranges of amino acid length (*x*), box horizontal lines correspond to 25‐, 50‐ and 75‐percentiles, whiskers for 5‐ and 95‐percentiles and a rectangle for mean. (c) BUSCO analysis of genomes “‐g” and proteomes “‐p” of the three vouchers: *Boletus subvelutipes* NYSf3039 (assembly JAMFLD000000000), *B. subvelutipes* MG31 (assembly ASM331603), *Boletus edulis* (assembly Boled5).

Gene prediction with BRAKER (Hoff et al., 2019) delivered 20,437 peptide sequences for the holotype NYSf3093 and 14,968 for strain MG31. As a control of our annotation pipeline, we used *B. edulis* assembly Boled5, for which 18,718 proteins are currently known; BRAKER identified 23,237 proteins for it. This difference indicates the presence of false‐positive gene candidates, about 20% of the Boled5‐braker outcome. We analyzed the distribution of protein lengths and concluded that, despite the fact that high degree of genome fragmentation in NYSf3093 increased the number of short proteins, we can work with this data. The average peptide length was: 356 amino acids for *B. edulis* (472 amino acids identified in Boled5‐braker), 367 amino acids for strain MG31, and 278 amino acids for the holotype NYSf3093 (Figure 6a,b). The resulting coding sequences were evaluated in BUSCO v.5.4.4 (boletales_odb10) to show that Boled5‐genome is 92% complete (its “proteome” is 93.6% complete and Boled5p‐braker is 92.5% complete). These parameters were 77% for genome (72.8% for proteome) for MG31 and 66.3% for genome (66.6% for proteome) for the holotype NYSf3093 (Figure 6c).

Fungi‐specific carbohydrate‐active enzymes (CAZymes) reflect habitat‐related substrate utilization (which may be composed of plant cell wall cellulose, pectin, lignin, etc.) and are an inherent characteristic of the fungal lifestyle. The portfolio of metabolic enzymes is an essential feature in the evolutionary speciation process and a relevant factor contributing to the taxonomy of fungi (Barrett et al., 2020). Analysis of CAZymes using dbCAN2 meta‐server revealed 469 CAZymes in strain MG31 and 473 in the holotype NYSf3093. Boled5 genome encodes 539 CAZymes (Boled5‐BRAKER—898). The number of secreted proteins in the CAZymes group may be related to the ability to break down their respective substrates. Thus, via the BRAKER annotation, we identified a set of secretory pathway proteins from a group of fungal CAZymes whose functional pattern resembles that of known fungal genomes such as *B. edulis*. This result proves that our annotation worked, and the genes in a group of typically secreted enzymes indeed possess the signal peptide that marks these proteins to be destined towards the secretory pathway.

**FIGURE 7:**
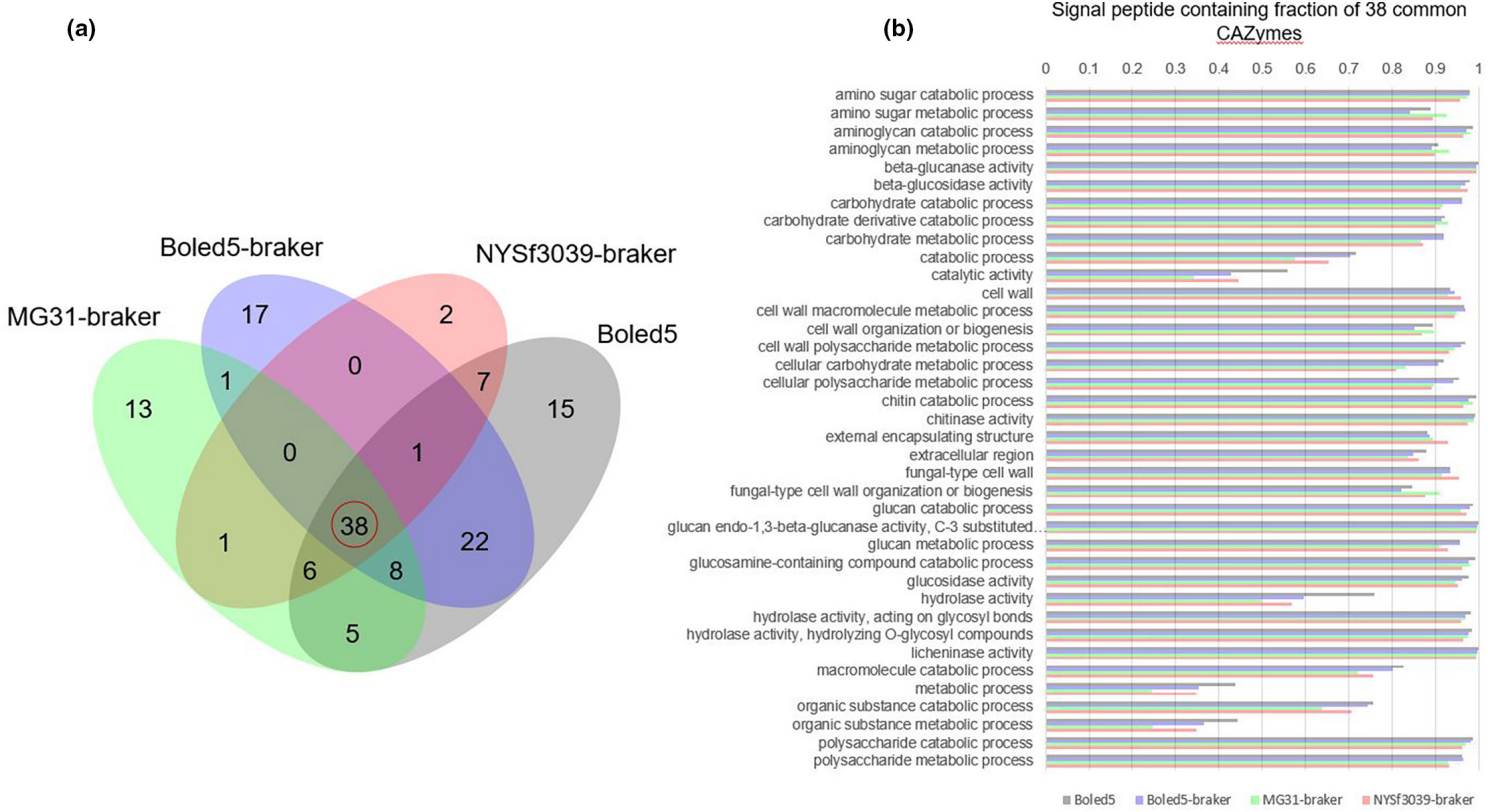
Venn diagrams of CAZymes (a) and SignalP containing fraction of 38 CAZymes with signal peptide bar chart of enrichment analysis for reduced overrepresented GOs groups (*p*‐value < .001) (b). Boled5—*Boletus edulis* BED1 genome, NYSf3093—*Boletus subvelutipes* NYSf3093 assembly JAMFLD000000000, MG31—*B. subvelutipes* strain MG31 assembly ASM331603.

We also ran an analysis of proteins containing signal peptides of external export with SignalP v.6 (Teufel et al., 2022), allowing us to examine the composition of GO (gene ontology) annotations using Fisher's exact test (comparing a group of CAZymes with a signal peptide vs. all proteins of the group). GO functional annotations were slightly different for NYSf3093 and strain MG31 (Figure 7). Thirty‐eight common to all gene ontology CAZymes are shown in Figure 7. It shows, for example, that almost all proteins with licheninase activity contain a signal peptide in all four genomes.

### 3.3 Phylogeny

**FIGURE 8:**
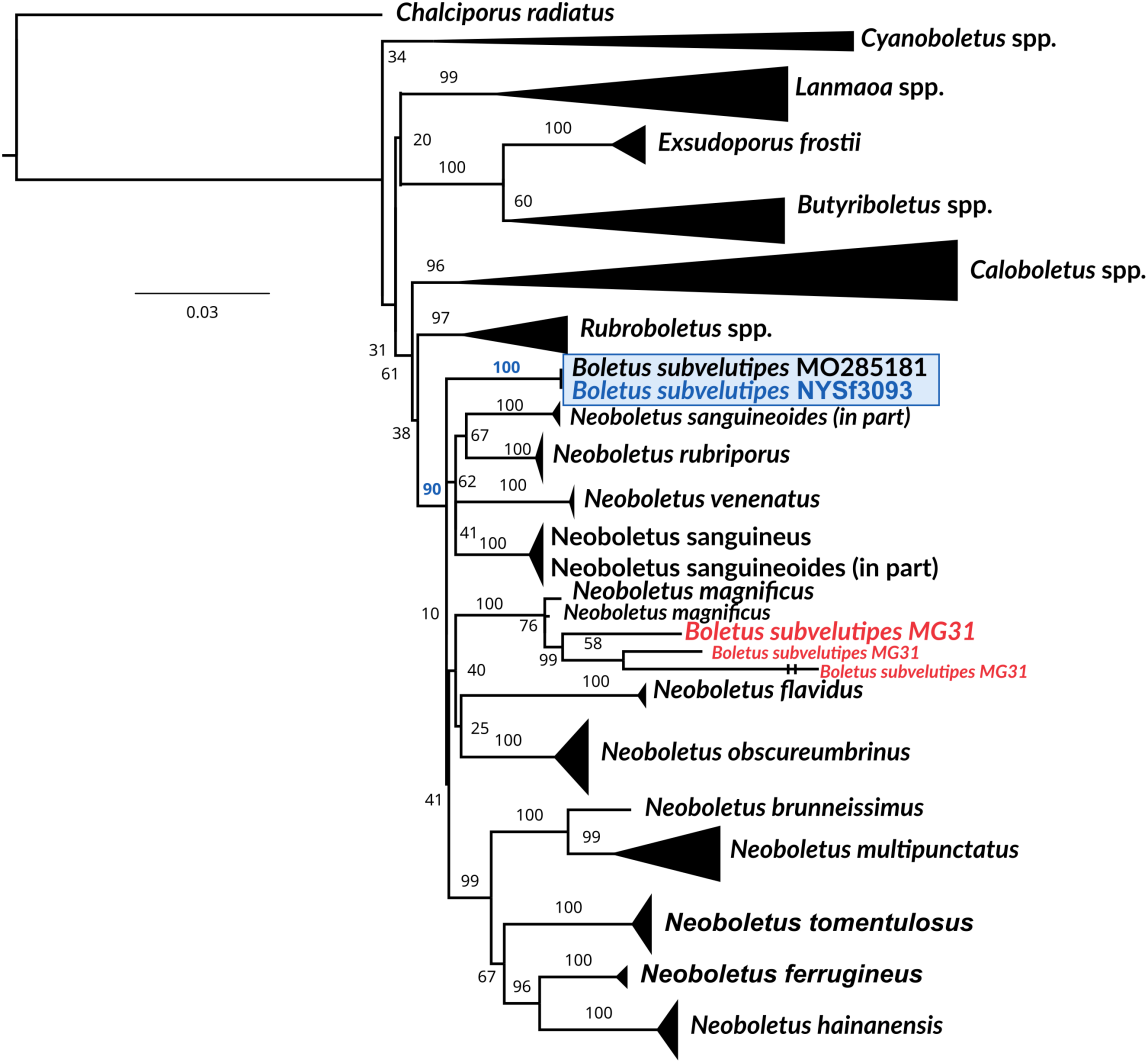
Phylogenetic relationships among representative species of Boletaceae inferred from TEF1, RPB2, ITS, and 28S using Maximum Likelihood with 1000 bootstraps. The bootstrap frequencies are shown on the supported branches. Blue rectangle: clade containing *Boletus subvelutipes* NYSf3093. Red text: the clade containing *B. subvelutipes* MG31.

To study the relationships between *B. subvelutipes* Peck voucher NYSM:NYSf3093 and “*Boletus subvelutipes* strain MG31” in more detail, we constructed a multilocus phylogenetic tree using TEF1, ITS, RPB2, and 28S sequences. These genomic sequences from our assembly have been separately uploaded to NCBI GenBank under accession numbers OP503538, OQ680486, and ON142311. Figure 8 shows a multilocus‐based Maximum Likelihood phylogenetic tree from a concatenated matrix. The named species and clades in the tree are all members of the “Pulveroboletus Group” (Wu et al., 2014, 2016), a large assemblage of bolete genera that are not part of the currently known 6 subfamilies within Boletaceae, as defined by Wu et al. (2016). Our holotype specimen resolves with *B. subvelutipes* voucher MO285181 (Figure 1b) with zero branch length and maximum node support, suggesting these two are conspecific. This clade, in turn, is placed as part of a large clade consisting of *Neoboletus* spp., with high statistical support (90%). The specimen identified as *B. subvelutipes* MG31 in (Li et al., 2018) is represented by several leaves due to alternative sequences of 28S and ITS on different scaffolds, possibly representing assembly errors or base‐call errors. These sequences were found forming a clade with *Neoboletus magnificus*, with 100 UFB. Our phylogeny suggests that *B. subvelutipes* strain MG31 is not identical to *B. subvelutipes* NYSf3093 and likely represents another species, *N. magnificus* (*=Sutorius magnificus*, *Boletus magnificus*), endemic to China.

Our conclusion to distinguish “strain MG31” and voucher of the holotype NYSf3093 from each other at the species level is also supported by the fact that the former voucher was purchased at a local fresh market by researchers from Kunming University, China (Li et al., 2018). Most boletes are host‐specific mycorrhizal fungi restricted to narrow geographic areas (Chai et al., 2019; Nuhn et al., 2013; Ortiz‐Santana et al., 2007; Urban & Klofac, 2015; Wu et al., 2016). For example, the bolete taxa of the eastern and western US, by and large, do not overlap (Bessette et al., 2010; Frank et al., 2020; Siegel & Schwarz, 2016). *Boletus subvelutipes* is known to be mycorrhizal primarily with eastern hemlock, *Tsuga canadensis*, a conifer native to eastern North America. Hence, its natural distribution in China is unlikely.

In summary, the genomic sequence of Peck's holotype from this study clarifies the taxonomic and phylogenetic disposition of *B. subvelutipes* in Boletaceae, solidifies its identity through comparison to the DNA of modern *B. subvelutipes* voucher, and obviates the need for epitypification (Hyde & Zhang, 2008). The “manual” method of DNA isolation and WGS procedure are suggested as an optimized way to obtain DNA data from limited amounts of fungal historical herbarium specimens.

## AUTHOR CONTRIBUTIONS

**Maria Shumskaya:** Conceptualization (equal); investigation (equal); methodology (equal); project administration (equal); supervision (equal); writing – original draft (equal); writing – review and editing (equal). **Kirill S. Mironov:** Formal analysis (equal); investigation (equal); methodology (equal); software (equal); validation (equal); visualization (equal); writing – original draft (equal). **Jesús A. Ballesteros:** Formal analysis (supporting); investigation (equal); software (equal); visualization (supporting); writing – original draft (supporting); writing – review and editing (supporting). **Igor Safonov:** Conceptualization (lead); resources (equal); supervision (equal); validation (equal); writing – original draft (equal); writing – review and editing (equal). **Roy E. Halling:** Conceptualization (lead); resources (equal); supervision (lead); validation (equal); writing – original draft (supporting); writing – review and editing (supporting).

## Supporting information


Data S1.

## Data Availability

The raw data were deposited at www.ncbi.nlm.nih.gov as Sequence Read Archive (SRR18532845, SRR18532846 for “manual” and “kit” DNA isolations) under BioProject PRJNA821397 and BioSample SAMN27069427. *Boletus subvelutipes* (Peck) NYSf3093 assembly—JAMFLD000000000, mitochondrial rRNA sequences—ON142312, nrDNA region containing 28S, 5.8S, 18S rRNA and ITS1 and ITS2 sequences—ON142311, TEF1—OP503538, RPB2—OQ680486.
